# DONSON facilitates Cdc45 and GINS chromatin association and is essential for DNA replication initiation

**DOI:** 10.1093/nar/gkad694

**Published:** 2023-08-28

**Authors:** Georgia Kingsley, Aggeliki Skagia, Paolo Passaretti, Cyntia Fernandez-Cuesta, Alicja Reynolds-Winczura, Kinga Koscielniak, Agnieszka Gambus

**Affiliations:** Institute of Cancer and Genomic Sciences, Birmingham Centre for Genome Biology, University of Birmingham, UK; Institute of Cancer and Genomic Sciences, Birmingham Centre for Genome Biology, University of Birmingham, UK; Institute of Cancer and Genomic Sciences, Birmingham Centre for Genome Biology, University of Birmingham, UK; Institute of Cancer and Genomic Sciences, Birmingham Centre for Genome Biology, University of Birmingham, UK; Institute of Cancer and Genomic Sciences, Birmingham Centre for Genome Biology, University of Birmingham, UK; Institute of Cancer and Genomic Sciences, Birmingham Centre for Genome Biology, University of Birmingham, UK; Institute of Cancer and Genomic Sciences, Birmingham Centre for Genome Biology, University of Birmingham, UK

## Abstract

Faithful cell division is the basis for the propagation of life and DNA replication must be precisely regulated. DNA replication stress is a prominent endogenous source of genome instability that not only leads to ageing, but also neuropathology and cancer development in humans. Specifically, the issues of how vertebrate cells select and activate origins of replication are of importance as, for example, insufficient origin firing leads to genomic instability and mutations in replication initiation factors lead to the rare human disease Meier-Gorlin syndrome. The mechanism of origin activation has been well characterised and reconstituted in yeast, however, an equal understanding of this process in higher eukaryotes is lacking. The firing of replication origins is driven by S-phase kinases (CDKs and DDK) and results in the activation of the replicative helicase and generation of two bi-directional replication forks. Our data, generated from cell-free *Xenopus laevis* egg extracts, show that DONSON is required for assembly of the active replicative helicase (CMG complex) at origins during replication initiation. DONSON has previously been shown to be essential during DNA replication, both in human cells and in *Drosophila*, but the mechanism of DONSON’s action was unknown. Here we show that DONSON’s presence is essential for replication initiation as it is required for Cdc45 and GINS association with Mcm2–7 complexes and helicase activation. To fulfil this role, DONSON interacts with the initiation factor, TopBP1, in a CDK-dependent manner. Following its initiation role, DONSON also forms a part of the replisome during the elongation stage of DNA replication. Mutations in *DONSON* have recently been shown to lead to the Meier-Gorlin syndrome; this novel replication initiation role of DONSON therefore provides the explanation for the phenotypes caused by *DONSON* mutations in patients.

## Graphical Abstract

**Graphical Abstract:**
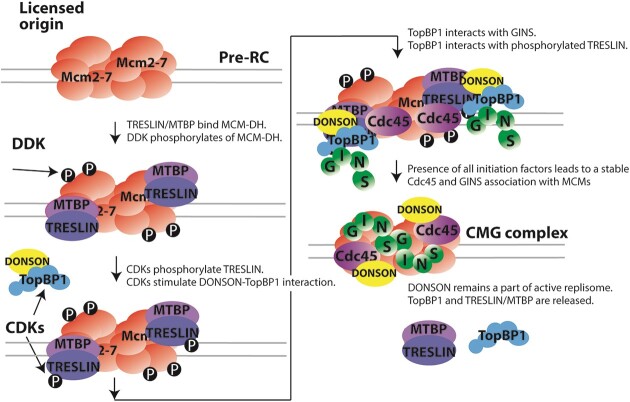

## INTRODUCTION

Faithful cell division is the basis for the propagation of life. The replication, repair and epigenetic regulation of the human genome are three of nine interrelated pathways involved in ageing (1,2) and defects in the maintenance of genome integrity are the foremost drivers of cellular deterioration (2). The mechanisms of origin firing during DNA replication initiation and replication fork progression have been well characterised and reconstituted in yeast, however, equal understanding of these processes in higher eukaryotes is lacking. Specifically, the issue of how vertebrates, including humans, select and activate origins of replication are not resolved conclusively and are of importance as, for example, areas with a paucity of replication origins are more prone to genomic instability (3), while mutations in factors involved in origin firing lead to the rare human disease Meier-Gorlin syndrome (4).

**Figure 1.**
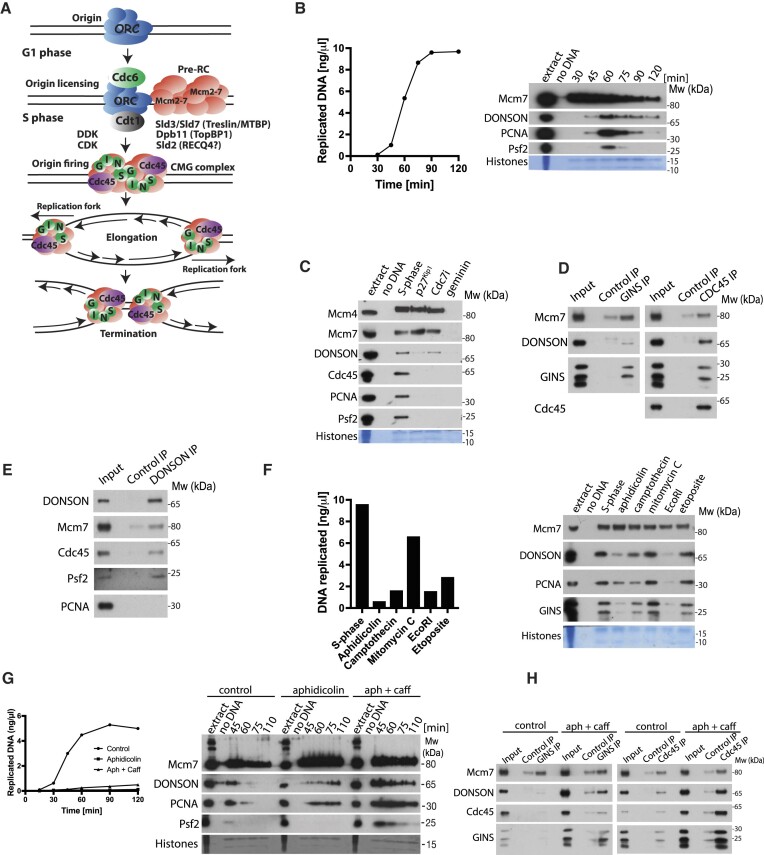
DONSON interacts with replisome on replicating chromatin in *Xenopus laevis* egg extract. (**A**) Model of eukaryotic DNA replication. *S. cerevisiae* initiation factors are listed with human homologues names in brackets. (**B**) DNA replication was set up in *Xenopus* egg extract and synthesis of nascent DNA followed by incorporation of α^32^P-dATP into newly synthesised DNA (left). At the same indicated times chromatin was isolated and chromatin bound factors resolved on SDS-PAGE and immunoblotted with indicated antibodies (right). ‘No DNA’ control served as chromatin specificity control, while Coomassie stained histones from the bottom of the gel serve as a loading and purity control. (**C**) Replication reaction was set up in egg extract in optional presence of indicated replication initiation inhibitors. Chromatin fractions were isolated in the middle of S-phase and analysed by immunoblotting as in (B). (**D**) Chromatin replicating in egg extract was isolated in the middle of S-phase, chromatin bound protein complexes were released from chromatin by digestion of DNA with benzonase and replisomes immunoprecipitated using α-Cdc45 or α-GINS specific antibodies. Non-specific commercial IgG antibodies served as non-specific control. Input and immunoprecipitated samples were resolved on SDS-PAGE and immunoblotted with indicated antibodies. (**E**) Chromatin was prepared as in (D), but immunoprecipitation was carried out with α-DONSON antibody. (**F**) Replication reaction was set up in egg extract and treated optionally with indicated replication stress and DNA damaging agents. Chromatin was isolated after 60 min of reaction progression (middle/late stage of replication) and immunoblotted with indicated antibodies as in (B) (right), while the ability of the extract to synthesise nascent DNA was assessed after 90 min of replication reaction progression (left). (**G**) Replication reaction was set up in egg extract and optionally supplemented with aphidicolin and caffeine, as indicated. Progression of DNA synthesis during replication reaction was analysed (left), while chromatin bound factors were analysed by immunoblotting as in (B) (right). (**H**) Chromatin replicating in egg extract in optional presence of aphidicolin and caffeine was isolated, chromatin protein complexes released by DNA digestion and Cdc45 or GINS immunoprecipitated as in (D).

DNA replication occurs in three stages: initiation, elongation and termination. DNA replication initiates from thousands of replication origins. These are the positions within the genome where replicative helicases become activated and start unwinding DNA while moving in opposite directions, away from each other, creating two DNA replication forks. The replicative helicase is composed of Cdc45, the Mcm2–7 hexamer and the GINS complex (CMG complex) (5); it is positioned at the tip of replication forks and forms a platform for replisome assembly (6). Activation of the CMG helicase (initiation stage) is divided into two cell cycle stages (Figure 1A): loading of the inactive core of Mcm2–7 in the form of double hexamers in the G1 stage of the cell cycle (origin licensing) and subsequent activation of a proportion of the double hexamers by Cdc45 and GINS during S-phase (origin firing). Once established, the replication forks replicate chromatin (elongation stage) until they encounter forks, moving in an opposing direction, which initiated from neighboring origins (Figure 1A). At this point, the termination of replication forks takes place.

During replication initiation, double hexamers of Mcm2–7 are first phosphorylated by Drf1/Dbf4-dependent Cdc7 kinase (DDK) (7,8) (Figure 1A). In *Saccharomyces cerevisiae*, DDK phosphorylation of Mcm2–7 promotes the recruitment of Sld3/Sld7 and Cdc45 to origins. This is followed by S-phase cyclin-dependent kinase (S-CDK) phosphorylation of Sld3 and Sld2, which supports the formation and recruitment to chromatin of the pre-landing complex (formed of Dpb11, Sld2, GINS and DNA Polϵ), allowing for CMG helicase assembly (9,10) (Figure 1A). DDK-dependent MCM phosphorylation is reversed by protein phosphatase 1 (PP1), which is targeted to chromatin by Rif1 (11–13). In higher eukaryotes, Treslin/MTBP complex is homologous to Sld3/Sld7 and TopBP1 protein to Dpb11. Both Treslin/MTBP and TopBP1 were shown to be essential for the loading of Cdc45 and GINS onto the Mcm2–7 hexamer in higher eukaryotes (14–18). In contrast, the functional homologue of Sld2 is still under debate. The N-terminus of RecQ4/RecQL4 helicase (*X.l*. RTS) contains a region of homology to Sld2 and RecQ4 is essential for initiation of DNA replication (19,20). However, in the *X. leavis* egg extract system, which provided much of this pioneering research, immunodepletion of RecQ4 does not affect the binding of Cdc45 and GINS to Mcm2–7. Rather, RecQ4 was found to promote a later stage of conversion of the pre-initiation complex (20–24).

Mutations in *DONSON* lead to microcephalic primordial dwarfism (MPD), which is a collective term for a group of human disorders characterised by intra-uterine and postnatal growth delay alongside marked microcephaly (4,25). DONSON was shown to interact with the replisome and is proposed to protect stalled DNA replication forks from the action of nucleases (25). Cells with siRNA downregulated DONSON (siDONSON) exhibit accumulation of stalled and asymmetric DNA replication forks and generation of double-stranded DNA breaks, which can be partially rescued by co-depletion of nucleases that can act on stalled forks (25). Moreover, DONSON was shown to be important for proper activation of the S-phase checkpoint response (25). Intriguingly, DONSON was shown to interact with only a subset of replisomes—specifically ones that were activated in early S-phase rather than late S-phase (26). During early S-phase, DONSON was shown to help replication forks traverse inter-strand crosslinks (ICL) (26). Interestingly, the *Drosophila* homologue of DONSON, *humpty dumpty* (*hd*), was also found to be essential for DNA replication, especially in early cleavage divisions of fly embryos, as *hd* knockout embryos arrested during the first few nuclear cleavage cycles with incomplete chromosome segregation (27,28). This suggests the evolutionary conservation of DONSON importance for cell proliferation in the developing embryo.

Altogether, we know several processes during DNA replication that DONSON is involved in and the consequences of DONSON’s downregulation, however, the mechanisms by which DONSON delivers its functions and how they are regulated are unknown. We have therefore set out to determine the molecular mechanism of DONSON’s action during DNA replication in the *Xenopus laevis* egg extract system, which is the only higher eukaryotic cell-free system able to undertake a full round of cell-cycle regulated DNA replication. We have found that *X.l*. DONSON indeed interacts with replisomes during DNA replication but surprisingly, we found that DONSON is required for initiation of DNA replication – more specifically for the activation of replicative helicase by loading of GINS and Cdc45.

## MATERIALS AND METHODS

### Inhibitors

MLN4924 (A01139, Active Biochem) was dissolved in DMSO at 5 mM and added to the extract 15 min after addition of sperm chromatin at 10 μM. NMS873 (17674, Cayman Chemical Company) was dissolved in DMSO at 10 mM and added to the extract 15 min after addition of sperm nuclei at 50 μM. Caffeine (C8960, Sigma) was dissolved in water at 100 mM and added to the extract along with demembranated sperm chromatin at 5 mM. Aphidicolin was dissolved in DMSO at 8 mM and added to the extract along with demembranated sperm chromatin at 40 μM. EcoR1 (R6011, Promega) was purchased at stock 12 U/μl and added to the extract at 0.05 U/μl. Mitomycin C (Calbiochem) was dissolved in water at 5 mM in water and used at 500 μM. Etoposide (Calbiochem) was dissolved in DMSO at 10 mM and used at 200 μM. Camptothecin was dissolved in water at 10 mM and used at 500 μM. Cdc7i PHA-767491 was dissolved in water and added at 100 μM.

### Recombinant proteins

pET28a-DONSON, pET28a-DONSON-ΔLoop(P334-L410), pET28a-DONSON-ΔN(A2-G155), pET28a-DONSON-M463T, pET28a-DONSON-E521K, pET28a-DONSON-F171S, pET28a-DONSON-P230S and pET28a-DONSON-R217C vectors were used for protein expression in 2 l of BL21 (DE3) (1 mM IPTG was added at OD_600_ = 0.6, followed by incubation overnight at 18°C). Frozen bacterial pellets were lysed in resuspension buffer (500 mM NaCl, 50 mM NaH_2_PO_4_, 2 mM MgCl_2_, 10% glycerol, pH 9, 1 μg/ml of each: aprotinin, leupeptin and pepstatin) and supplemented with 1 mg/ml lysozyme, 0.05% Tween-20 and BitNuclease. After sonication, the lysate was clarified by centrifugation at 14 000 g for 30 min at 4°C, and supernatants containing soluble proteins were then incubated with 2 ml of pre-washed Super Ni-NTA Affinity Resin (SUPER-NINTA100, Generon) for 2 h, rotating at 4°C. The beads were then washed 3× with 30 ml of Lysis buffer, both supplemented with 15 mM imidazole. The beads were transferred to 10 ml columns (Poly-Prep Chromatography Column, Bio-Rad) and eluted with Lysis Buffer supplemented with 250 mM imidazole. Most concentrated elution fractions were dialysed against LFB1/50 buffer (10% sucrose, 50 mM KCl, 40 mM HEPES pH 8.0, 20 mM K phosphate pH 8, 2 mM MgCl_2_, 1 mM EGTA, 2 mM DTT, 1 μg/ml of each: aprotinin, leupeptin and pepstatin) and snap frozen in liquid nitrogen. Recombinant p27(KIP1) was described previously (8) and used at 100 nM, while geminin^DEL^ was described in (29).

### Antibodies

α-PCNA (P8825) and α-HIS (H1029) were purchased from Sigma; α-RIF1 (A300-568A) was purchased from Bethyl; α-PP1α (sc-271762) and α-PP2α (sc-56950) were purchased from Santa Cruz Biotechnology.

Affinity purified α-Cdc45, α-Psf2 (30), α-Mcm4 antibodies (31), α-Mcm7 (32), α-RecQ4 and α-TopBP1 antibodies were previously described (33). Similarly, α-GINS (34), α-Treslin and α-MTBP were described in (18).

*Xenopus* full-length DONSON protein was purified as described above and antibodies raised against such prepared antigens in sheep or rabbit. The resulting antibody sera was purified in-house against the purified antigen. The specificity of each new antibody is presented in Supplementary Figure S1.

### *Xenopus laevis* egg extract preparation

All of the work with *Xenopus laevis* was approved by Animal Welfare and Ethical Review Body (AWERB) at University of Birmingham and approved by UK Home Office in form of Project License issued for Dr Agnieszka Gambus. *Xenopus laevis* egg extract was prepared as previously described (35).

### Immunodepletion

DONSON immunodepletions with sheep antibodies were performed using Dynabeads Protein G (10004D, Life Technologies) coupled to antibodies against DONSON or nonspecific sheep IgGs (I5131, Sigma), with two rounds of 45 min incubation at 4°C. The DONSON antibodies were coupled at 600 μg per 1 ml of beads. Effective immunodepletion required two rounds of 45 min incubation of egg extract with antibody coupled beads at 50% beads ratio.

DONSON immunodepletions with rabbit antibodies were performed using Dynabeads Protein A (10008D, Life Technologies) coupled to *Xenopus* DONSON antibodies raised in rabbit and affinity purified or nonspecific rabbit IgG (I5006, Sigma). The DONSON antibodies were coupled at 600 μg per 1 ml of beads. Effective immunodepletion required two rounds of 45 min incubation of egg extract with antibody coupled beads at 50% beads ratio.

TopBP1 immunodepletions were performed using Dynabeads Protein G (10004D, Life Technologies) coupled to antibodies against TopBP1 or nonspecific sheep IgGs (I5131, Sigma). The TopBP1 antibodies were coupled at 600 μg per 1 ml of beads. Effective immunodepletion required two rounds of 45 min incubation of egg extract with antibody coupled beads at 50% beads ratio.

### DNA synthesis assay

Interphase *X. laevis* egg extract was supplemented with 10 ng/μl of demembranated sperm chromatin and incubated at 23°C for indicated time. Synthesis of nascent DNA was then measured by quantification of α^32^P-dATP (PerkinElmer) incorporation into newly synthesised DNA, as described before (35). The extract contains endogenous dNTP pools of ∼50 μM (36). The total amount of DNA synthesized, expressed as ng DNA / μl extract, can then be calculated by multiplying percent total ^32^P incorporated by a factor of 0.654 (36). This calculation assumes an average molecular weight of 327 Da for dNMPs and equal quantities of all four dNTPs incorporated into DNA (weight of dNMP incorporated in ng/μl = percent total ^32^P incorporated/100 × 50 × 10^−6^ × 4 × 327 × 10^3^) (36).

For quantification of replication efficiency between different experimental repeats in different extracts, the quantity of DNA replicated at the end of the reaction in IgG-depleted extract was set as 100% and the remaining values normalised to this.

### Chromatin isolation time-course

Interphase *X. laevis* egg extract was supplemented with 10–15 ng/μl of demembranated sperm DNA and subjected to indicated treatments. The reaction was incubated at 23°C for indicated length of time and chromatin was isolated in ANIB100 buffer (50 mM HEPES pH 7.6, 100 mM KOAc, 10 mM MgOAc, 2.5 mM Mg-ATP, 0.5 mM spermidine, 0.3 mM spermine, 1 μg/ml of each aprotinin, leupeptin and pepstatin, 25 mM β-glycerophosphate and 10 mM 2-chloroacetamide (Merck)) as described previously (35).

During the chromatin isolation procedure, a sample without addition of sperm DNA (no DNA) is processed in an analogous way, usually at the end of the time course, to serve as a chromatin specificity control. The bottom of the PAGE gel on which the chromatin samples were resolved is cut off and stained with Colloidal Coomassie (SimplyBlue, Life Technologies) to stain histones which provide loading controls and indications of sample contamination with egg extract (cytoplasm).

### Western blot quantification

The quantification of western blots is provided to indicate reproducibility of trends in experiments rather than to provide absolute values of increases or decreases in a signal. The quantified experiments were performed in different preparations of extracts and independently immunodepleted extracts to confirm that observed phenotypes are not specific for one extract prep.

The density of pixels of each band of the western blot and scanned Coomassie stained histones within the gel were quantified using Image J software. The numeric value in arbitrary units for each western blot band was normalised to appropriate loading control (bands of Coomassie stained histones from the same sample). The analysis of IgG-depleted (control) and treatment samples was always done together and the fold difference between them calculated. Fold change from three repeated experiments is plotted on the graph with mean value and standard error of the mean (SEM) as calculated by GraphPad PRISM.

### Immunoprecipitation from chromatin

275 μl of egg extract per IP was induced into interphase and mixed with 10–15 ng/μl demembranated sperm nuclei and optionally supplemented with the indicated treatments. The reaction was incubated at 23°C for the indicated time. Chromatin was isolated in ANIB100 (50 mM HEPES pH 7.6, 100 mM KOAc, 10 mM MgOAc, 2.5 mM Mg-ATP, 0.5 mM spermidine, 0.3 mM spermine, 1 μg/ml of each aprotinin, leupeptin and pepstatin, 25 mM β-glycerophosphate, 0.1 mM Na_3_VO_4_, 0.1% triton and 10 mM 2-chloroacetamide), and the chromatin pellets re-suspended in the same volume of original extract of ANIB100 containing 20% sucrose. Protein complexes were released from chromatin by digestion with 2 U/μl of Benzonase nuclease (E1014-25KU, Sigma) and sonicated for 5 min using a Diagenode sonicator with settings: 30 s on, 30 s off, medium setting. The insoluble fraction was then spun in a microfuge at 4°C, 10 min, 16k g.

Prepared beads:

- 30 μl of Dynabeads M-270 epoxy (14302D, Life Technologies) coupled covalently to 20 μg of affinity purified DONSON antibodies or IgG from sheep serum (I5131, Sigma);
- 100 μl of Dynabeads Protein A (10008D, Life Technologies) covalently coupled to 20 μg of affinity purified DONSON, affinity purified Treslin, or IgG from rabbit serum (I5006, Sigma) using BS3 crosslinker (S5799, Sigma);

were incubated with 220 μl digested chromatin at 4°C for 1–2 hours with rotation. Following the incubation time, beads were washed for 5 min rotating at 4°C twice with ANIB100, once with ANIB100 containing an additional 0.1% Triton X-100 and finally twice with ANIB100 buffer. Each sample was prepared by boiling in 50 μl of 2x NuPAGE LDS loading buffer (Life Technologies) for 5 min.

### Immunoprecipitation from nucleoplasm

550 μl of egg extract per IP was induced into interphase and mixed with 10–15 ng/μl demembranated sperm chromatin and optionally supplemented with the indicated treatments. CDK inhibitor (p27^Kip1^) and CDC7 inhibitor (PHA-767491) were added immediately after sperm DNA. The reaction was incubated at 23°C for 45 min. Nucleoplasm was isolated in ANIB100 (50 mM HEPES pH 7.6, 100 mM KOAc, 10 mM MgOAc, 2.5 mM Mg-ATP, 0.5 mM spermidine, 0.3 mM spermine, 1 μg/ml of each aprotinin, leupeptin and pepstatin, 25 mM β-glycerophosphate and 10 mM 2-chloroacetamide), and the nuclear pellets re-suspended in the same volume of original extract of ANIB100 containing 20% sucrose and 0.1% triton. Protein complexes were released from nucleoplasm by digestion with 2 U/μl of Benzonase nuclease (E1014-25KU, Sigma) and sonicated for 5 min using a Diagenode sonicator with settings: 30s on, 30s off, medium setting. The insoluble fraction was then spun in a microfuge at 4°C, 10 min, 16 000 rcf.

100 μl of Dynabeads Protein A (10008D, Life Technologies) covalently coupled to 20 μg of affinity purified DONSON or IgG from rabbit serum (I5006, Sigma) using BS3 crosslinker (S5799, Sigma); were incubated with 400 μl digested nucleoplasm at 4°C for 1–2 h with rotation. Following the incubation time, beads were washed for 5 min rotating at 4°C twice with ANIB100, once with LFB1/50 and finally twice with ANIB100 buffer. Each sample was prepared by boiling in 50 μl of 2× NuPAGE LDS loading buffer (Life Technologies) for 5 min.

### Large scale immunoprecipitation of DONSON for mass spectrometry and CHROMASS

3.9 ml of *X. laevis* egg extract was activated and supplemented with 10 ng/μl of demembranated sperm DNA, 50 μM p97 inhibitor NMS873 or 40 μM aphidicolin + 5 mM caffeine and incubated at 23°C for 60 min. Chromatin was isolated in ANIB/100 buffer. Immunoprecipitation of DONSON was performed as described previously for p97 (37) and the immunoprecipitated material was analysed by mass spectrometry with Dr Richard Jones from MS Bioworks LLC.

Similarly, chromatin isolated from 10 μl of egg extract was processed in analogous way for mass spectrometry.

### Sample preparation

Each sample was run on a 5–20% gradient gel (Invitrogen) for 1 cm and cut into 10 bands. Samples were submitted pre-plated for ten fraction analysis. Gel pieces were processed using a robot (ProGest, DigiLab) with the following protocol:

- Washed with 25 mM ammonium bicarbonate followed by acetonitrile.
- Reduced with 10 mM dithiothreitol at 60°C followed by alkylation with 50 mM iodoacetamide at RT.
- Digested with trypsin (Promega) at 37°C for 4 h.
- Quenched with formic acid and the supernatant was analysed directly without further processing.

### Mass spectrometry

The gel digests were analysed by nano LC/MS/MS with a Waters M-class HPLC system interfaced to a ThermoFisher Oribitrap Fusion Lumos. Peptides were loaded on a trapping column and eluted over a 75 μm analytical column at 350 nl/min; both columns were packed with Luna C18 resin (Phenomenex). A 30 min gradient was employed. The mass spectrometer was operated in data-dependent mode, with MS and MS/MS performed in the Orbitrap at 60 000 resolution and 15 000 resolution, respectively. Advanced Peak Detection was turned on. The instrument was run with a 3 s cycle for MS and MS/MS. Proteome Discoverer v1.4 was used for peak generation.

### Data processing

Data were searched using Mascot (Matrix Science, London, UK; version 2.8.0.1) with the following parameters:

Enzyme: Trypsin Fully Specific
Database: Uniprot Xenopus (forward and reverse appended with common contaminants) released on 15 April 2014. 79 274 (including reverse and CON) entries in the database were searched.
Fixed modification: Carbamidomethyl (C)
Variable modifications: Oxidation (M), Acetyl (Protein N-term), Deamidation (NQ), GlyGly (K), Phospho (STY)
Mass values: Monoisotopic
Peptide Mass Tolerance: 10 ppm
Fragment Mass Tolerance: 0.02 Da
Max Missed Cleavages: 2

Mascot DAT files were parsed into the Scaffold (version Scaffold_5.1.0, Proteome Software Inc., Portland, OR) software for validation, filtering and to create a non-redundant list per sample. Data were filtered with 1% protein and peptide false discovery rate (FDR) and requiring at least two unique peptides per protein.

Peptide identifications were accepted if they could be established at greater than 34.0% probability to achieve an FDR <1.0% by the Percolator posterior error probability calculation (38). Protein identifications were accepted if they could be established at greater than 99.0% probability to achieve an FDR less than 1.0% and contained at least two identified peptides. Protein probabilities were assigned by the Protein Prophet algorithm (39). Proteins that contained similar peptides and could not be differentiated based on MS/MS analysis alone were grouped to satisfy the principles of parsimony.

For calculation of fold enrichment for proteins with 0 peptides detected in control immunoprecipitation, that number was changed to 1 to allow for fold enrichment calculation.

### AlphaFold analyses

AlphaFold (AF) modelling was carried out using the AF-multimer software (40,41) installed on BlueBEAR, the high-performance computing (HPC) cluster at the University of Birmingham. *Xenopus laevis* DONSON protein structures were predicted using the standalone version of AlphaFold (2.1.1-foss-2021a-CUDA-11.3.1). The AlphaFold models used were selected after producing five conformations ranked by their per-residue confidence scores (pLDDT) for each conformation (42). Differently, Human DONSON AF structure was downloaded from UniProt. UniProt protein sequences used are Q5U4U4 and Q9NYP3 for *Xenopus* and human respectively. Molecular graphics and analyses performed with UCSF ChimeraX, (43,44).

## RESULTS

### DONSON interacts with replisomes on chromatin in egg extract

DONSON was previously reported to interact with replisomes in human cells, especially those activated in early S-phase (25,26) and we have found DONSON interacting with terminated replisomes on chromatin in *Xenopus* egg extract (Supplementary Figure S1A). In this experiment, (described previously in (37)), a DNA replication reaction was set up in the egg extract, supplemented with inhibitors to block unloading of ubiquitylated terminated replisomes: cullin E3 ubiquitin ligases inhibitor (MLN4924) to block Cul2^Lrr1^ from ubiquitylating Mcm7 within the terminated CMG helicases, and an ATPase-dead mutant of p97 segregase (p97mut). Such treatments led to the accumulation of large quantities of post-termination replisomes on chromatin. They were immunoprecipitated with Mcm3 antibodies and co-immunoprecipitated proteins were analysed by mass spectrometry (37). Many replisome components were identified this way and, interestingly, we also found multiple peptides of DONSON. As DONSON was reported to protect stalled replication forks in human cells (25), we hypothesised that it may also play a role in regulation of replisome disassembly during DNA replication. Therefore, we raised two antibodies, in sheep (α-DONSONs) and in rabbit (α-DONSONr) against recombinant *X.l*. DONSON purified from bacteria (Supplementary Figure S1B). We used these antibodies to analyse the pattern of DONSON chromatin binding during DNA replication in *Xenopus* egg extract.

Firstly, we analysed an unperturbed DNA replication reaction (Figure 1B). This reaction was initiated in egg extract by addition of the substrate DNA (demembranated sperm chromatin), and the progress of the replication reaction followed by incorporation of α^32^P-dATP into the synthesised nascent DNA was measured in parallel to isolation of the chromatin bound proteins for western blot analysis (Figure 1B). As expected, we could observe Mcm7 binding to chromatin from the beginning of the reaction as origin licensing takes place within the first 5 min of the replication reaction in our system. The replisome components were present on chromatin at 60 min, which coincides with the fast rate of DNA synthesis in this experiment and thus a large number of replication forks replicating DNA. Finally, we could observe DONSON binding to chromatin at a similar time as the replisome factors but found that it was retained at a lower level for a longer time (Figure 1B). We decided therefore to test whether the observed DONSON interaction with chromatin depends on active replication (Figure 1C). For this, we inhibited DNA replication either through inhibition of origin licensing with geminin (Mcm4 and Mcm7 not present on chromatin), or through inhibition of CDK activity with p27^Kip1^ (Mcm4 loaded and phosphorylated by DDK; no Cdc45, PCNA and Psf2 loading), or through inhibition of DDK activity with Cdc7i PHA-767491 (Mcm4 loaded but not phosphorylated; replication fork factors not present). In all situations where the DNA replication reaction was inhibited, we observed reduced binding of DONSON to chromatin (Figure 1C), suggesting that DONSON’s interaction with chromatin is mostly dependent on active replication. The reduction of binding was reproducibly less profound upon Cdc7 inhibition suggesting that DONSON’s chromatin interaction depends more on CDK activity. We verified also that once on chromatin during S-phase, DONSON indeed forms part of the replisome, as it can co-immunoprecipitate with GINS (Psf2) and Cdc45 from a replicating chromatin fraction (Figure 1D and E).

As discussed previously, we observed DONSON interacting with immunoprecipitated terminated replisomes (Supplementary Figure S1A). To follow up this observation, we analysed DONSON chromatin binding when replisome unloading was inhibited either by inhibition of Cul2^Lrr1^ activity using MLN4924 neddylation inhibitor (Supplementary Figure S1D) or by inhibiting the activity of p97 segregase with the NMS973 inhibitor (Supplementary Figure S1C). In both cases, we observed that DONSON accumulates on chromatin with similar timings as terminated replisomes. Altogether, these data suggest that DONSON continues interacting with replisomes at later stages of the replication.

As DONSON was reported in human cells to be important for cell survival when challenged with replication stress, and to fully activate S-phase checkpoint (25,26), we next analysed whether DONSON’s chromatin binding activity is stimulated by different forms of replication stress and DNA damage (Figure 1F). In this case, the DNA replication reactions were set up in the presence of different stressors: aphidicolin—inhibitor of replicative polymerases; camptothecin – inhibitor of Topoisomerase 1, causing ssDNA breaks and DNA–protein crosslinks (DPCs); mitomycin C (MMC)—causing interstrand crosslinks (ICLs); EcoRI – restriction enzyme inducing double-stranded DNA breaks (DSBs); etoposide—inhibitor of Topoisomerase 2, causing DSBs and DPCs. All these treatments reduced the treated extract's ability to synthesise nascent DNA, albeit to different levels, and showed differing levels of reduction in replisome components binding to replicating chromatin. In all treatments, DONSON’s chromatin binding pattern was most similar to that of other replisome components (PCNA and GINS), and we did not observe stimulation of DONSON chromatin association upon any of the treatments (Figure 1F).

To learn more about DONSON’s interaction with chromatin upon replication stress and S-phase checkpoint activation, we also set up replication reactions in the presence of aphidicolin alone or with aphidicolin and caffeine together. Caffeine is a potent ATM/ATR inhibitor and in the presence of aphidicolin and caffeine, replication forks fire and stall due to inhibition of polymerases; however, as the checkpoint is not active, origins fire uncontrollably leading to an accumulation of high numbers of stalled replication forks on chromatin, while very little nascent DNA is being synthesised (Figure 1G). Importantly, under these conditions we can observe that DONSON accumulates on chromatin together with the stalled replication forks, suggesting that S-phase checkpoint activity is not needed for DONSON chromatin binding.

Finally, we wanted to determine whether DONSON still interacts with replisomes upon S-phase checkpoint inhibition. To achieve this, we isolated replicating chromatin challenged with aphidicolin/caffeine as above, digested DNA to release protein complexes from chromatin and immunoprecipitated GINS complex or Cdc45 (Figure 1H). Reassuringly, we observed that DONSON co-immunoprecipitated with GINS and Cdc45 together with other replisome factors, irrespectively of checkpoint inhibition. Comparing DONSON interactions with the replisome components on normal S-phase chromatin and chromatin from aphidicolin/caffeine treated extract, we can detect the same interactions, although there are more replisomes and more DONSON accumulated upon aphidicolin/caffeine treatment and thus we detect their interactions more robustly.

### DONSON is essential for replication in *Xenopus* egg extract

**Figure 2.**
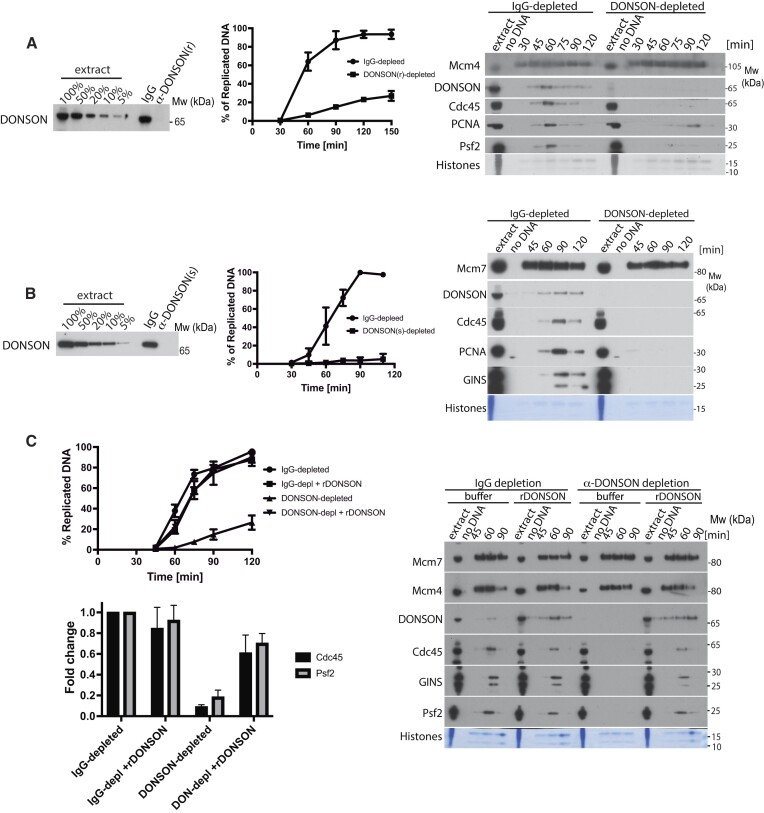
DONSON is essential for DNA replication in *X.laevis* egg extract. (**A**, **B**) *Xenopus* egg extract was immunodepleted of DONSON using either rabbit α-DONSON antibodies (**A**) or sheep α-DONSON antibodies (**B**). Nonspecific rabbit or sheep commercial IgGs were used for control IgG– depletions. The efficiency of depletion was established by immunoblotting in comparison to titration of original extract (left). The ability of immunodepleted extracts to synthesise nascent DNA was analysed by incorporation of α^32^P-dATP into newly synthesised DNA (middle). Mean of *n* = 5 for rabbit DONSON depletion, *n* = 3 for sheep DONSON depletion is presented with SEM. The binding of replication fork factors in the absence of DONSON was analysed by immunoblotting of isolated chromatin fractions as in Figure 1B (right). (**C**) Replication reaction was set up in IgG- or DONSON-depleted extracts with optional addition of recombinant wt DONSON. The level of nascent DNA synthesis was followed as above (left top, *n* = 3, Mean with SEM is presented) and chromatin was isolated at indicated times and analysed by immunoblotting with indicated antibodies as in (A) (right). Quantification of fold change of chromatin bound Cdc45 and Psf2 at the peak of replication in IgG- and DONSON-depleted extract with optional addition of rDONSON over *n* = 3. The levels were normalised to level in IgG-depleted extract. Mean with SEM (left, bottom).

Having established that DONSON is a component of normal replisomes in the *Xenopus* system, we aimed to determine the importance of DONSON for replication in the egg extract. To this end, we immunodepleted DONSON from egg extract using each of the raised antibodies. Both antibodies were able to immunodeplete DONSON to a level lower than 5% of DONSON remaining in the extract (Figure 2A, B). We then analysed the ability of IgG-depleted and DONSON-depleted extracts to synthesise nascent DNA. In both cases, the replication capacity of DONSON-depleted extract was very strongly diminished (Figure 2A, B). We also analysed chromatin binding of DNA replication factors in IgG- and DONSON-depleted extracts and could observe no DONSON, but also no Cdc45 and GINS binding to chromatin in the DONSON-depleted extract, which went hand in hand with the inhibition of DNA synthesis (Figure 2A, B). Importantly, we could rescue both the DNA synthesis and Cdc45 and GINS chromatin biding in DONSON-depleted extract through addition of recombinant *X.l*. DONSON purified from bacteria (Figure 2C and Supplementary Figure S1E). This rescue confirms that the only essential replication factor depleted in DONSON-depleted extract was DONSON itself. Addition of recombinant DONSON to DONSON-depleted egg extract readily rescues the levels of DNA synthesis in the extract, but fewer replication forks seem to be established to support this DNA synthesis. It is likely that DONSON purified from bacteria lacks important posttranslational modifications and is not as active as the endogenous DONSON.

### DONSON is essential for replication initiation

**Figure 3.**
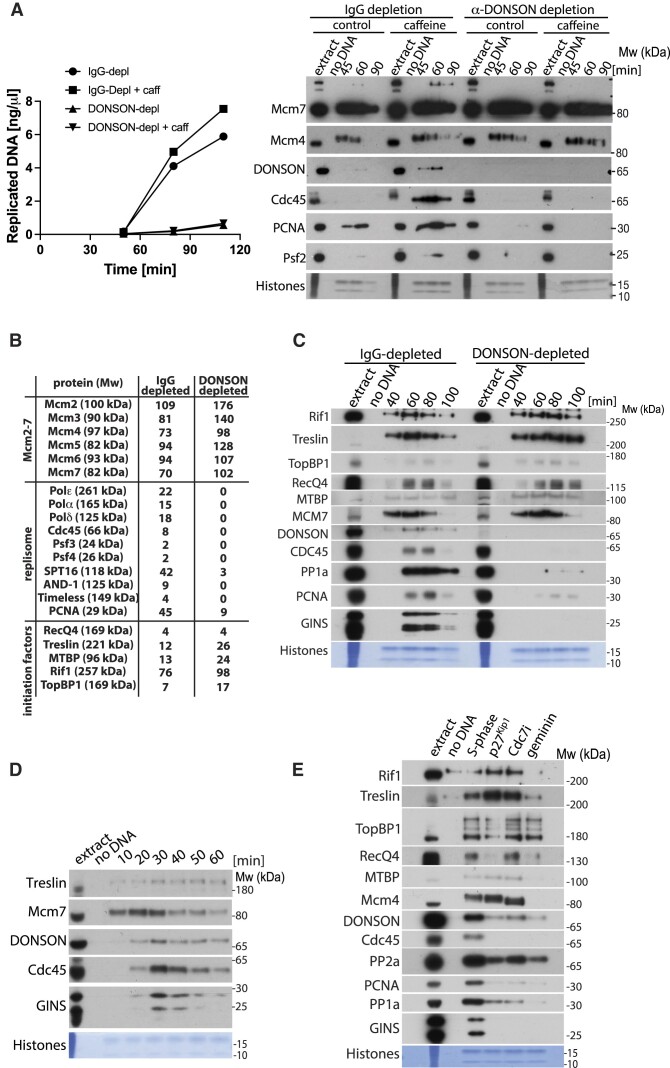
DONSON is essential for replication initiation in egg extract. (**A**) Replication reaction was set up in IgG- or DONSON-depleted egg extract optionally supplemented with caffeine. DNA synthesis was analysed throughout the reaction by incorporation of α^32^P-dATP into newly synthesised DNA (left) and chromatin bound factors analysed at indicated times with indicated antibodies (right). (**B**) Egg extract was immunodepleted of DONSON with rabbit antibodies as in Figure 2A, chromatin was isolated in the middle of S-phase (45 min) and whole chromatin proteome of IgG- and DONSON-depleted reaction analysed by mass spectrometry. The total spectral count of selected replication factors is presented. (**C**) Replication reaction was set up in IgG- or DONSON-depleted egg extract and chromatin isolated at indicated times. Chromatin bound factors were analysed by immunoblotting with indicated antibodies as in Figure 1B. (**D**) Replication reaction was set up in *Xenopus* egg extract, chromatin isolated at indicated times during early stages of replication reaction and analysed as above. (**E**) Replication reaction was set up in egg extract in optional presence of indicated replication initiation inhibitors. Chromatin fractions were isolated in the middle of S-phase and analysed by immunoblotting as in (A).

As DONSON was proposed to stabilise stalled replication forks in human cells (25), we next tested whether the inhibition of DNA replication in DONSON-depleted extract is due to the collapse of early replication forks, which could lead to S-phase checkpoint activation and inhibition of any further origin firing. To do so, we supplemented IgG- or DONSON-depleted extract with caffeine to block potential checkpoint activation (Figure 3A). Such treatment of DONSON-depleted extract did not rescue its ability to synthesise DNA, and importantly, did not rescue Cdc45 and GINS chromatin binding (Figure 3A) suggesting that it is not checkpoint activation that inhibits Cdc45 and GINS chromatin binding in DONSON-depleted extract. We also verified that DONSON depletion is not blocking the formation of the nuclear envelope around chromatin in the egg extract, which is essential for replication in our system (45) (data not shown).

During DNA replication initiation, origins are first licensed by loading of Mcm2–7 double hexamers onto chromatin. Mcm2–7 are then phosphorylated by DDKs and the CDKs to stimulate Cdc45 and GINS association through modification of several initiation factors. Our analyses of chromatin in DONSON-depleted extracts show that the origin licensing stage happens normally as we observe normal levels of chromatin-bound components of Mcm2–7 complexes (Figure 2). Moreover, analysis of chromatin-bound Mcm4 in DONSON-depleted extract revealed the same levels of phosphorylation as in IgG-depleted extract, suggesting that the DDK phosphorylation step is executed with similar efficiency in the absence of DONSON (Figure 2A and C, Mcm4). To understand, therefore, how DONSON functions to support DNA replication in the egg extract we first analysed by mass spectrometry the proteome of replicating chromatin in IgG- and DONSON-depleted extracts (CHROMASS) (Figure 3B). While we observed a complete absence of replisome components (Cdc45, GINS, polymerases, AND-1 etc.) in the DONSON-depleted extract, we also observed increased levels of Mcm2–7 components, presumably as they are not unloaded from chromatin due to inhibition of replication. Interestingly, we also observed higher levels of some of the initiation factors Treslin/MTBP complex and TopBP1 (Figure 3B), suggesting that the replication reaction is inhibited in DONSON-depleted extract at the stage of initiation. We confirmed these findings by western blotting in IgG- and DONSON-depleted extracts (Figure 3C), showing that without DONSON initiation factors like: Rif1, Treslin/MTBP, TopBP1 and RecQ4 all remain bound to chromatin throughout the time course of the reaction, while the replisome components are missing (Figure 3C).

### DONSON’s cooperation with other replication initiation factors

As DONSON seems to be involved in the initiation stage of DNA replication, we next examined in more detail the timings of chromatin association for DONSON and other initiation factors (Figure 3D). We set up an unchallenged DNA replication reaction in the egg extract, but this time analysed a pattern of protein binding to chromatin early on during the reaction, before onset of replication forks firing. As shown previously (Figure 1B), we can see binding of Mcm7 from the earliest time point (10 min) due to rapid origin licensing. Treslin starts binding to chromatin early (10–20 min), which is consistent with a previous report showing that the Treslin/MTBP complex binds chromatin before replication initiation (18). In this replication reaction, Cdc45 and GINS start binding chromatin at 20 min with a peak at 30–40 min. DONSON’s pattern of chromatin binding mostly resembles that of Cdc45 and GINS (Figure 3D).

We also compared chromatin binding of DONSON and other initiation factors upon inhibition of replication initiation at different stages of the reaction: inhibition of licensing (geminin treatment), inhibition of DDK activity (PHA-767491 Cdc7i) and inhibition of CDK activity (p27^Kip1^ inhibitor) (Figure 3E). Treatment with geminin was shown previously to significantly reduce chromatin binding of Treslin/MTBP and RecQ4 (18,20,46), while inhibition of CDK activity with p27^Kip1^ led to Treslin/MTBP hyper-loading onto chromatin (18,46) and partially reduced loading of RecQ4 (18,20,46). Inhibition of DDK activity was shown to have a less profound of an effect on Treslin/MTBP chromatin binding (18). Our observations agree with these previous findings. Previous studies concerning TopBP1 however, showed that chromatin binding was either unaffected by all such treatments (47) or reduced to different levels by all of them (18,46). In our hands, we observe that TopBP1 chromatin binding is partially reduced after CDK inhibition. Some of these differences are likely to be the effect of different antibodies used in different studies. Interestingly, DONSON chromatin binding is reduced upon inhibition of S-phase kinases DDK and CDK, especially with the latter, and its chromatin binding pattern resembles much more the replisome components rather than other initiation factors (Figure 3E), suggesting that it is acting relatively late in the initiation cascade.

**Figure 4.**
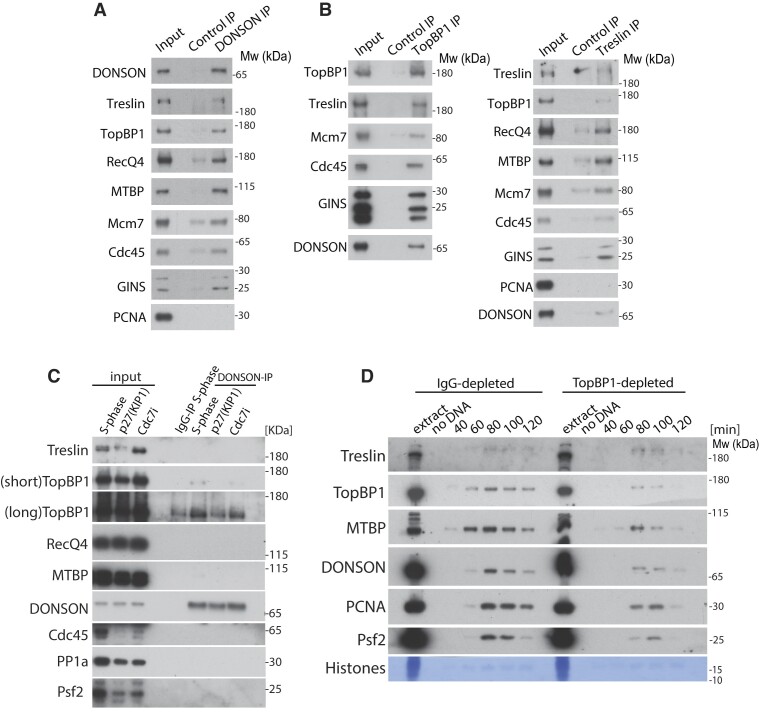
DONSON interacts with replication initiation factors. (**A**) Chromatin replication reaction was carried on in presence of aphidicolin and caffeine. Chromatin was isolated after 60 min of replication reaction, protein complexes were released from chromatin by digestion of DNA with benzonase and non-specific control antibodies or α-DONSON(s) antibodies used for immunoprecipitation. The input and immunoprecipitated samples were analysed by immunoblotting with indicated antibodies. (**B**) As (A) but α-TopBP1 or α-Treslin antibodies were used for immunoprecipitation. (**C**) DNA replication reaction was set up in egg extract with optional addition of replication initiation inhibitors (p27^KIP1^ or Cdc7i). In the middle of S-phase, replicating nuclei were isolated in a buffer without detergent, which allows to retain part of the nucleoplasm. From this input material, DONSON was immunoprecipitated and immunoprecipitation samples analysed by immunoblotting with indicated antibodies. As a control, non-specific antibody immunoprecipitation from unchallenged S-phase input was performed in parallel. (**D**) TopBP1 was immunodepleted from egg extract to 25% of the original level. Replication reaction was set up in IgG- and TopBP1-depleted extract and chromatin isolated at indicated times of reaction and chromatin fractions analysed as above.

In an effort to understand the mechanism by which DONSON exerts its initiation function, we first compared interactors of DONSON on chromatin at early or late replication stages. To do so, we accumulated DONSON and other replisome components on chromatin either at the termination stage, by inhibiting replisome unloading with p97i (akin to the experiment in Supplementary Figure S1C), or at an earlier stage of replication by supplementing extract with aphidicolin and caffeine (akin to Figure 1F); here, the replisomes are stalled, but new origins keep firing due to checkpoint inhibition. We immunoprecipitated DONSON and analysed co-immunoprecipitated factors by mass spectrometry. We then calculated the enrichment of replisome and replication initiation factors in both IPs (Supplementary Figure S2A). Interestingly, we observed enrichment of peptides of TopBP1 and RecQ4 in the DONSON IP from aphidicolin/caffeine treated sample. We then analysed interactions between DONSON and initiation factors within the aphidicolin/caffeine chromatin fraction by immunoblotting (Figure 4A). Here we observed that DONSON interacts with the replisome components, as we have seen in Figure 1, but interestingly also with TopBP1, RecQ4 and Treslin/MTBP (Figure 4A). Importantly, reciprocal immunoprecipitation of TopBP1 and Treslin from aphidicolin/caffeine treated chromatin fraction shows analogous interactions with DONSON (Figure 4B). Altogether these data suggest again that DONSON is not only a part of the mature replisome, but also plays a role at the initiation stage.

### DONSON interacts with TopBP1

We have shown that DONSON forms a part of the replisome during the initiation stage of DNA replication as it can interact with replication initiation factors on chromatin (Figure 4A and B). We next wanted to understand whether any of these interactions can occur directly with DONSON, outside the context of a replisome. We therefore isolated nuclei containing nucleoplasm (chromatin and nucleoplasm), in a buffer without addition of detergent, and immunoprecipitated DONSON. Interestingly, out of all potential interacting proteins tested, DONSON could interact with TopBP1 and this interaction was strongly reduced by p27^Kip1^ CDK inhibitor (Figure 4C). We therefore partially depleted TopBP1 from egg extract to 25% of its original concentration (Supplementary Figure S2B) and analysed whether chromatin binding of DONSON depends on TopBP1 (Figure 4D). With this, we observed that the level of chromatin-bound DONSON was strongly reduced. Altogether, all these data suggest that DONSON interacts with chromatin in a TopBP1- and CDK-dependent manner and acts as a replication initiation factor, essential for Cdc45 and GINS recruitment to the CMG complex.

### DONSON’s protein structure and mutations

**Figure 5.**
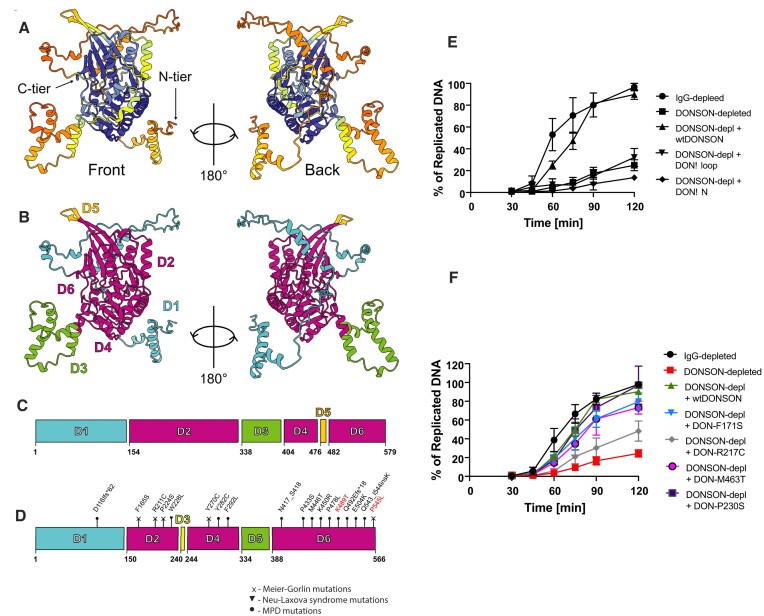
DONSON 3D structure organisation. (**A**) AlphaFold prediction of DONSON 3D structure. High confidence prediction in blue, low confidence prediction in yellow/orange. (**B, C**) Based on the predicted structure DONSON can be divided into six domains—N-terminal D1 domain which is unstructured, structured D2, flexible loop D3, structured D4 and D6 with a short turn motif D5. The 1D model of DONSON with colour coded domains (C) and 3D representation of these domains separation (B). (**D**) Mapping of the disease mutations described in (48) on human DONSON structure. Meier-Gorlin, Microcephalic Primordial Dwarfism MPD syndrome and Neu-Laxova syndrome mutations are mapped. The mutations marked in red do not have *Xenopus* homologous sequence. (**E**) DONSON mutant lacking unstructured N-terminus (DONSONΔN) or the flexible loop D3 from (B), (DONSONΔloop), was purified (Supplementary Figure 3C) and tested for rescue of DONSON-depleted egg extract in its ability to synthesise nascent DNA. (**F**) Indicated point mutants of DONSON (*Xenopus* DONSON with mutations homologous to patient derived human mutations) were purified (Supp Figure 3C) and tested as in (E).

We next wanted to explore whether DONSON’s function may be conserved through evolution. The AlphaFold prediction of *X.l*. DONSON’s structure suggests that DONSON is formed of a long, mostly unstructured N-terminus (D1), wrapping around a folded globular core with one protruding flexible loop (D3) (Figure 5A–C). Interestingly, the folded globular core of DONSON is highly conserved throughout evolution, while the unstructured N-terminus is not (Supplementary Figure S3A and B) and the flexible loop is partially conserved. We therefore wanted to examine whether this flexible parts of DONSON are important for its function. We purified DONSON without the N-terminus (DONSONΔN) or without the flexible loop (D3) (DONSONΔloop) (Supplementary Figure S3C) and tested whether they can rescue replication in the egg extract immunodepleted of DONSON. First, we titrated wt DONSON into DONSON-depleted extract to establish the minimal concentration needed for a DONSON rescue (Supplementary Figure S3D) and then tested the mutants (Figure 5E). Although both deletions generated proteins that were well expressed, soluble and could be easily purified (Supplementary Figure S3C), they could not rescue the ability of the DONSON-depleted extract to synthesise nascent DNA (Figure 5E).

We also mapped known patient mutations causing microcephalic primordial dwarfism (MPD), specifically Meier-Gorlin syndrome in humans (48), onto the model structure of human DONSON (Figure 5D). Interestingly, most of the Meier-Gorlin mutations cluster within the human DONSON D2 domain (corresponding to the first half of the *X.l*.DONSON D2 domain). To test the effect of DONSON disease-causing mutations on replication initiation, we cloned and purified a selection of DONSON point mutations that were reported to cause MPD or Meier-Gorlin syndrome in humans (Supplementary Figure S3C). We chose two mutations leading to MPD: M446T (*Xenopus* M463T), which is a homozygous mutation found in 3 patients, E504K (*Xenopus* E521K), which was found in 2 patients as a compound heterozygous mutation combined with a stop codon introducing mutation. Interestingly, we could not efficiently express *X.l*. DONSON-E521K in bacteria, most likely due to protein destabilisation (Supplementary Figure S3C), while *X.l*. DONSON-M446T purified well and could fully restore the ability of the DONSON-depleted extract to synthesise nascent DNA (Supplementary Figure S3C and Figure 5F). We also chose three mutations leading to Meier-Gorlin syndrome: R211C (*Xenopus* R217C), which is a homozygous mutation found in two patients, and F165S (*Xenopus* F171S), which was found in one patient as a compound heterozygous mutation combined with an intronic splice site mutation, and another compound heterozygous mutation P224S (*Xenopus* P230S). All three mutants were readily purified (Supplementary Figure S3C) and while *X.l*. DONSON-F171S and DONSON-P230S could rescue the ability of DONSON-depleted extract to synthesise nascent DNA, *X.l*. DONSON-R217C was partially defective (Figure 5F), suggesting that this mutation may disrupt the functionality of DONSON during replication initiation.

Altogether, these investigations revealed that flexible and poorly conserved fragments of DONSON are nevertheless essential for DONSON activity and that some of the patient mutations leading to the Meier-Gorlin syndrome phenotype can indeed disrupt the essential function of DONSON during DNA replication initiation.

## DISCUSSION

Based on the data presented here we would like to propose that DONSON is a novel DNA replication initiation factor, as it is clearly essential for loading of Cdc45 and GINS onto origins during initiation. In the absence of DONSON, the replicative helicase, the CMG complex, cannot be formed and without the helicase activity, replication forks cannot be assembled. Mechanistically, our data suggest that DONSON acts through direct interaction with TopBP1, and this interaction is dependent on CDK activity. TopBP1, and its yeast homologue Dpb11/Cut5, is a master regulator of replication initiation. TopBP1 it is thought to bridge together Treslin/MTBP in complex with Cdc45, and RecQ4 in complex with GINS (Figure 1A) (14–20). However, immunodepletion of RecQ4 from *Xenopus* egg extract does not inhibit Cdc45 and GINS chromatin binding (20), suggesting that RecQ4, despite the partial homology to Sld2, does not play an equivalent role in higher eukaryotes.

TopBP1 acts as a scaffold protein, able to make numerous protein-protein interactions, facilitated in most cases through the multiple BRCT domains. BRCT domains most often interact with phosphorylated partners and often act in pairs. Yeast Dpb11 (*S. cerevisiae*) and Cut5 (*S. pombe*) contain 4 BRCT domains each, with BRCT1 + 2 interacting with phosphorylated Sld3 (9) and BRCT3 + 4 interacting with phosphorylated Sld2 (49,50). However, the human and *Xenopus* TopBP1 homologues are much larger proteins, containing eight BRCT domains (1-8), with an additional N-terminal BRCT domain identified in human TopBP1, named BRCT0 (51). This evolution of multiple additional BRCT domains suggests that higher eukaryotic TopBP1 is likely to have additional interactors, functions and regulations than the yeast counterparts. Like the yeast protein however, TopBP1 binds with phosphorylated Treslin (at S1001 in human and S976 in *Xenopus*) through its BRCT1 + 2 domains (14). TopBP1 also contains a GINS interacting motif (GINI), which lies between BRCT3 and BRCT4 domains (52), and was proposed recently to interact directly with GINS using BRCT4 + 5 domains (53). Interestingly, although BRCT4 + 5 are homologous to fungal BRCT3 + 4, it is BRCT7 + 8 that binds RecQ4, but this interaction is not dependent on CDK phosphorylation in either species (21,54). Moreover, TopBP1 BRCT7 + 8 are not essential for replication initiation at all (46), strengthening the argument that RecQ4 cannot be the functional homologue of yeast Sld2.

AlphaFold prediction of the DONSON-TopBP1 interaction (not shown) suggests that DONSON interacts with BRCT3 of TopBP1. Interestingly, it was shown that although the N-terminal fragment of TopBP1 spanning BRCT1 + 2 is sufficient to interact with Treslin, it is not sufficient to support DNA replication in the *Xenopus* egg extract. Instead, the TopBP1 fragment including BRCT1-3 is essential and sufficient to support replication initiation (46). This suggests an essential initiation role for BRCT3 in TopBP1, which thus far was not assigned to interact with any specific initiation factor. DONSON could therefore be such a factor. Interestingly, DONSON’s interaction with TopBP1 is dependent on CDK activity, as would be expected for the functional homologue of Sld2. It remains to be determined however, whether this interaction is driven by direct phosphorylation of DONSON by CDKs, or perhaps phosphorylation of another factor.

We propose here that DONSON likely fills the role of a functional homologue of Sld2 in higher eukaryotes. In yeast, Dpb11-Sld2 forms a ‘pre-landing complex’ together with GINS and DNA Pol ϵ (55). In our analyses of DONSON interactions, we could detect DONSON interacting with GINS and DNA Pol ϵ, but only from chromatin fractions, where many interactions can be stimulated by formation of replisomes around the CMG complex. The only robust interaction that we found for DONSON within the nucleoplasmic fraction was that with TopBP1. It is not yet clear, therefore, whether DONSON and TopBP1 indeed form an analogous pre-landing complex. How else could DONSON act during origin firing to stimulate Cdc45 and GINS interaction with Mcm2–7? The molecular mechanism underpinning how TopBP1 and Treslin/MTBP deliver GINS and Cdc45 to Mcm2–7 remains unknown. It is clear from the work of many groups that Treslin/MTBP bind Mcm2–7 early in the initiation reaction. Their interaction is strengthened by DDK activity but does not require CDK activity; on the contrary, they are hyper-loaded upon inhibition of CDKs (18,46). Phosphorylation of Treslin/MTBP by CDKs stimulates their interaction with N-terminal BRCT repeats of TopBP1 and is essential for helicase activation (14–15,46). TopBP1 can interact with GINS and is clearly essential for Cdc45 and GINS chromatin binding (47,53). We show here that DONSON interaction with TopBP1 depends on CDK activity. It is likely therefore that upon CDK activation, both TopBP1 and DONSON are recruited to phosphorylated Treslin/MTBP at Mcm2–7 double hexamers. The presence of all these initiation factors together allows for stable recruitment of Cdc45 and GINS into a CMG complex and helicase activation. TopBP1 is a limiting factor for origin firing (56) and is likely to be released following helicase activation, to facilitate firing of further origins, while DONSON is likely to be retained as part of the replisome in *Xenopus* egg extract.

Our data clearly indicate that DONSON plays an essential role in initiation of DNA replication, but DONSON has been shown previously in human cells to regulate the progression of replication forks, especially in response to replication stress (25,26). However, the molecular function of DONSON has largely been investigated in highly unstable HeLa cells (25,26), which often overexpress replication factors to support fast proliferation. Moreover, DONSON was always downregulated using siRNA (which takes several days to efficiently downregulate the protein level) or through analysis of patient-derived mutants, which destabilise the protein and lead to continuous lower levels of DONSON (25,26). Finally, the effects of DONSON downregulation on DNA replication were analysed by DNA fibre analyses or creation of DNA damage, which would miss cells that do not initiate DNA replication. Therefore, it is possible that the initiation phenotype of DONSON downregulation had been missed thus far in human cells and awaits careful dissection and determination. Interestingly, DONSON was also proposed to specifically form part of early firing replisomes, and not the ones in late S-phase (26). This observation raises the possibility that DONSON specifically activates early firing origins, in contrast to Rif1, which is proposed to regulate firing of mainly late replication origins (57). Such a specified role for DONSON would also explain why it is so essential during embryogenesis in both *Xenopus* egg extract and in *Drosophila* embryos (27,28), as both of these exemplify embryonic systems, which have very fast DNA replication and lack replication timing programmes, so all origins can be regarded as early firing.

Downregulation of DONSON in human cells has been shown to diminish the ability of the fired replication forks to progress past replication obstacles and causes activation of S-phase checkpoint (25,26). We observed here that DONSON interacts with replisomes not only during replication initiation but also at later stages of the DNA replication reaction. DONSON accumulates with the rest of the replisome on chromatin upon inhibition of unloading of post-termination replisomes and interacts with post-termination replisomes. It is very likely therefore that after facilitating the initiation of replication machinery, DONSON travels with the replisomes and regulates fork progression. To study such a function of DONSON, we need to develop separation-of-function mutants, that can deliver the initiation function but are defective in the fork progression function. It is possible that some patient mutations may deliver such tools for the future.

Mutations in DONSON have been known for a while to be the underlying genetic problems, which lead to microcephalic primordial dwarfism (MPD). Interestingly, mutations in DONSON were also recently shown to specifically cause Meier-Gorlin syndrome (48,58), the subtype of MPD caused by dysregulation of DNA origin firing and replication initiation. The discovery of a replication initiation role for DONSON therefore explains the phenotypes caused by these mutations. It is interesting to speculate that if DONSON has multiple roles during DNA replication, different mutations within the protein can specifically disrupt these different functions. Most DONSON patient mutations have been shown to destabilise the protein and lead to much lower levels of DONSON protein in the cells (25). Nevertheless, it is possible that some point mutations (e.g. R211C) disrupt specifically its interaction with TopBP1, while others may destabilise interactions with other replication factors important for fork progression and checkpoint activation. More research is needed to answer all these questions.

Finally, in the last two years, researchers have reached a better understanding of the importance of DONSON in cancer development. DONSON was found to be a biomarker for risk stratification in clear cell renal carcinoma (ccRCC) (59), and in prostate carcinoma (PCa) (60). It has also been linked with a malignant phenotype in ccRCC cell culture models (61). Furthermore, it was shown to be a driver of breast cancer progression and a potential target for subsequent therapies (62). Understanding the different molecular functions of DONSON during DNA replication provides us with a solid foundation for translation of DONSON’s potential as a biomarker and as a therapy target in the future.

## Supplementary Material

gkad694_Supplemental_Files

## Data Availability

The mass spectrometry proteomics data have been deposited to the ProteomeXchange Consortium via the PRIDE repository with the dataset identifier: **Project Name:** Chromatin proteome of S-phase in IgG- and DONSON-depleted egg extract **Project accession**: PXD042435 **Project DOI:** 10.6019/PXD042435
