# Supplementary material for: DONSON facilitates Cdc45 and GINS chromatin association and is essential for DNA replication initiation
Source: Nucleic Acids Res. 2023 Aug 28;51(18):9748–63. doi: 10.1093/nar/gkad694 (PMC10570026; doi:10.1093/nar/gkad694)
Supplement: gkad694_Supplemental_Files [file gkad694_supplemental_files.zip › Supp figure 1.pdf]

A

	Control (IgG)	IP (Mcm3)
Mcm3 (90 kDa)	17 (20%)	1111 (84%)
Mcm2 (100 kDa)	17 (17%)	975 (73%)
Mcm4 (97 kDa)	7 (7%)	947 (80%)
Mcm5 (82 kDa)	30 (18%)	927 (84%)
Mcm7 (82 kDa)	24 (10%)	905 (77%)
Mcm6 (93 kDa)	39 (18%)	888 (83%)
Cdc45 (66 kDa)	2 (3%)	277 (54%)
Psf1 (23 kDa)	0	62 (85%)
Psf2 (21 kDa)	0	33 (85%)
Psf3 (24 kDa)	0	55 (92%)
Sld5 (26 kDa)	0	44 (64%)
Ctf4 (125 kDa)	6 (17%)	554 (54%)
Spt16 (118 kDa)	3 (3%)	454 (55%)
SSRP (79 kDa)	0	229 (51%)
Timeless (149 kDa)	0	171 (32%)
Tipin (40 kDa)	0	54 (26%)
Top2a (179 kDa)	6 (3%)	484 (51%)
Claspin (146 kDa)	0	72 (21%)
PolA1 (165 kDa)	0	109 (33%)
PolA2 (67 kDa)	0	7 (11%)
PolE1 (261 kDa)	2 (1%)	543 (40%)
PolE2 (60kDa)	0	103 (55%)
DONSON (64 kDa)	0	68 (32%)

B

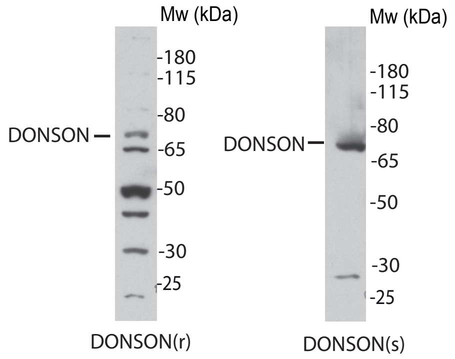

E

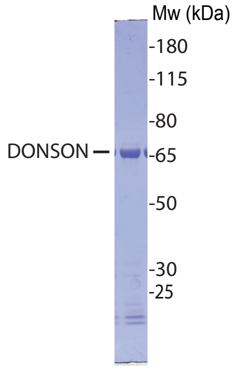

C

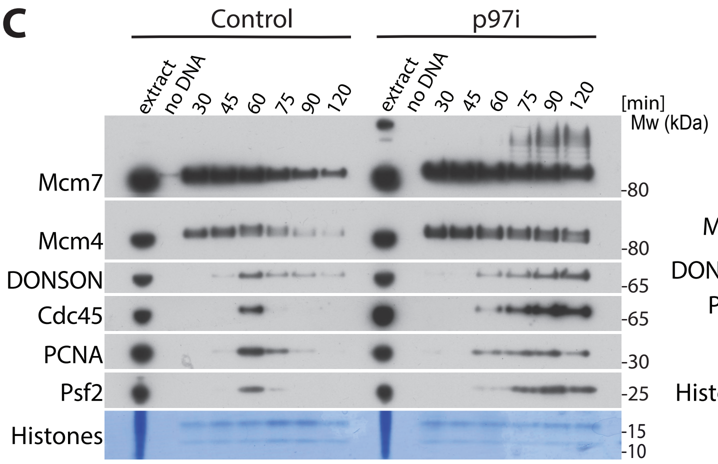

D

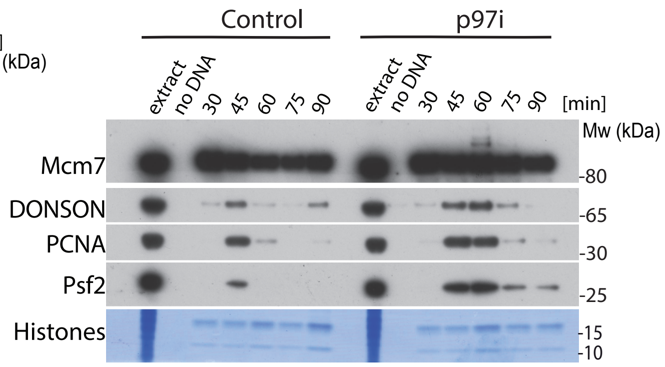

Supp Figure 1. DONSON interacts with terminated replisomes. **(A)** DNA replication reaction was established in Xenopus laevis egg extract supplemented with inactive mutant of p97 segregase and MLN4924 cullin E3 ligase inhibitor to block disassembly of replisomes in S-phase (as described in Sonneville et al 2017). Chromatin containing retained terminated replisomes was isolated, fragmented with benzonase and Mcm3 immunoprecipitaed. Proteins interacting with Mcm3 were analysed by mass spectrometry. Total spectral count and percentage coverage is presented for selected replisome components. **(B)** Polyclonal rabbit and shhep antibodies were raised against recombinant X.l.DONSON purified from bacteria. Protein band corresponding to DONSON within egg extract is ndicated. **(C)** Replicaton reaction was set up in egg extract supplemented with p97i NMS932 and chromatin isolated at indicated times. Chromatin samples were analysed by western blotting with indicated antibodies as in Figure 1. **(D)** As in (C) but the replication reaction was supplemented with cullin E3 ligase inhibitor MLN4924. **(E)** X.laevis 6HIS-DONSON expressed in bacteria and affinity purified.
