# Supplementary material for: DONSON facilitates Cdc45 and GINS chromatin association and is essential for DNA replication initiation
Source: Nucleic Acids Res. 2023 Aug 28;51(18):9748–63. doi: 10.1093/nar/gkad694 (PMC10570026; doi:10.1093/nar/gkad694)
Supplement: gkad694_Supplemental_Files [file gkad694_supplemental_files.zip › Supp Figure 2.pdf]

A

Fold enrichment in DONSON IP

	protein	p97i chromatin	aph/caff chromatin
	DONSON	84	86
Mcm2-7	Mcm2	1.3	1.7
Mcm2-7	Mcm3	1.4	2.8
Mcm2-7	Mcm4	1.5	2.9
Mcm2-7	Mcm5	1.3	4.7
Mcm2-7	Mcm6	1.3	1.4
Mcm2-7	Mcm7	1.2	3.4
CMG	Psf2	6	0
CMG	Psf4	2.3	0
CMG	Psf1	1.9	0
CMG	Cdc45	2.1	0
initiation factors	RecQ4	1.25	7
initiation factors	RPA	5	2.29
initiation factors	Treslin	2	0
initiation factors	MTBP	3.6	4
initiation factors	TOPBP1	0	7
initiation factors	Rif1	5.9	7

B

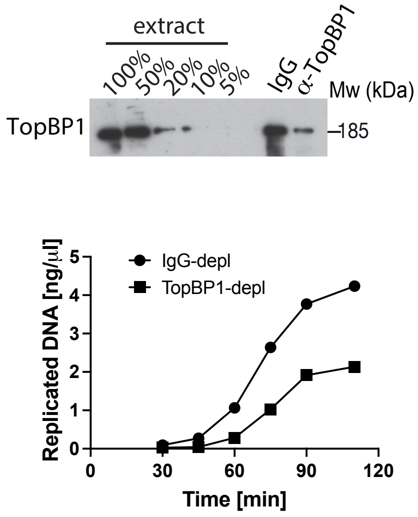

Supp Figure 2. (A) DNA replication was set up in egg extract either supplemented with p97i to accumulate post-termination replisome or with aohidicolin and caffeine to accumulate stalled and continously estabishing replisomes. Chromatin sample was isolated either in late (for p97 inhibitor, 90 min) or middle (for aphidicolin/caffeine, 60 min) S-phase, protein complexes released from chromatn by DNA digestion and non-specific control and DONSON(s) antibodies used for immunoprecipiatation. The fold enrichment of presented replication fators in DONSON IP over nonspecific conrol IP was coalculated and is preseneted. **(B)** TopBP1 was immunodepleted from egg extract. The efficiency of immunodepletion was analysed by western blotting against titration of original egg extract (top) and the ability of the immunodepleted extract to synthesise nascent DNA analysed by incorporation of α^{32}P-dATP into synthesised DNA (bottom).
