# Supplementary material for: DONSON facilitates Cdc45 and GINS chromatin association and is essential for DNA replication initiation
Source: Nucleic Acids Res. 2023 Aug 28;51(18):9748–63. doi: 10.1093/nar/gkad694 (PMC10570026; doi:10.1093/nar/gkad694)
Supplement: gkad694_Supplemental_Files [file gkad694_supplemental_files.zip › Supp Figure 3.pdf]

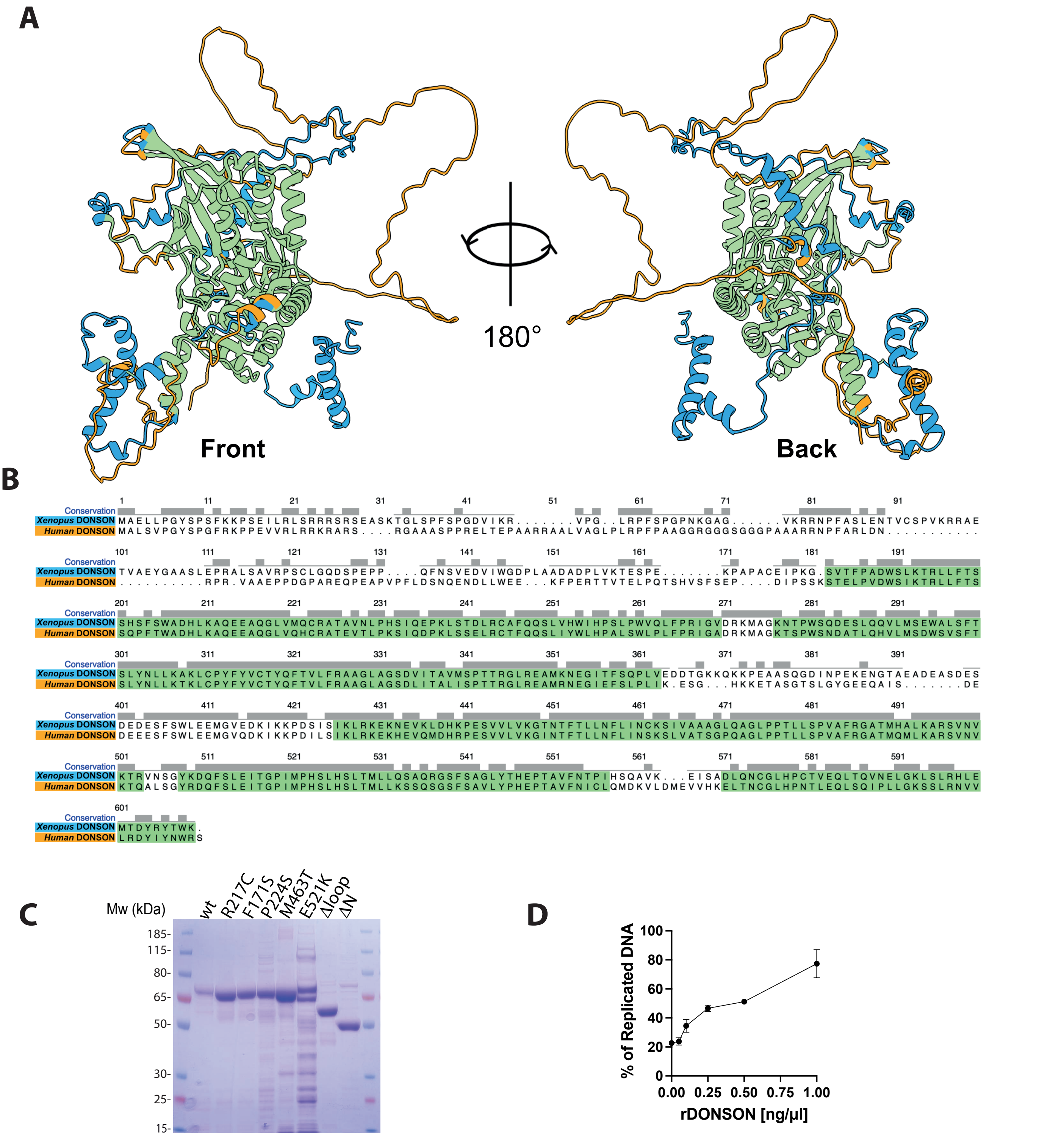

Supp Figure 3. Conservation of DONSON structure **(A)** AlphaFold overlay of 3D structure of Xenopus DONSON (blue) and human DONSON (orange). Overlapping structure in green. **(B)** Comparison of XenopusDONSON (blue) and human DONSON (orange) primary sequences. Conserved sequence in green. **(C)** Purification of DONSON truncation and patient mutations indicted. **(D)** Titration of recombinant wt DONSON into DONSON-depleted egg extract. The % of control replication level of IgG-depleted egg extract is presented as mean of n=2 with SEM.
